# An integrative approach for the identification of prognostic and predictive biomarkers in rectal cancer

**DOI:** 10.18632/oncotarget.4935

**Published:** 2015-09-02

**Authors:** Marco Agostini, Klaus-Peter Janssen, ll-Jin Kim, Edoardo D'Angelo, Silvia Pizzini, Andrea Zangrando, Carlo Zanon, Chiara Pastrello, Isacco Maretto, Maura Digito, Chiara Bedin, Igor Jurisica, Flavio Rizzolio, Antonio Giordano, Stefania Bortoluzzi, Donato Nitti, Salvatore Pucciarelli

**Affiliations:** ^1^ Department of Surgical, Oncological and Gastroenterological Sciences, Section of Surgery, University of Padova, Padua, Italy; ^2^ Istituto di Ricerca Pediatrica- Città della Speranza, Padua, Italy; ^3^ The Methodist Hospital Research Institute, Houston, USA; ^4^ Department of Surgery, UCSF, San Francisco, CA, USA; ^5^ Department of Surgery, UCSF, San Francisco, CA 94143, California, CA; ^6^ Department of Biology, University of Padua, Padua, Italy; ^7^ Department of Woman and Child Health, University of Padua, Padua, Italy; ^8^ Neuroblastoma Laboratory, Istituto di Ricerca Pediatrica- Città della Speranza, Padua, Italy; ^9^ Ontario Cancer Institute, the Campbell Family Institute for Cancer Research, and Techna Institute, University Health Network, Toronto, ON, Canada; ^10^ Department of Translational Research, National Cancer Institute – CRO-IRCSS, Aviano, Italy; ^11^ Sbarro Institute for Cancer Research and Molecular Medicine, Center for Biotechnology, College of Science and Technology, Temple University, Philadelphia, PA, USA; ^12^ Department of Molecular Medicine, University of Padua, Padua, Italy

**Keywords:** rectal cancer, integrated approach, biological network, prognostic, predictive

## Abstract

**Introduction:**

Colorectal cancer is the third most common cancer in the world, a small fraction of which is represented by locally advanced rectal cancer (LARC). If not medically contraindicated, preoperative chemoradiotherapy, represent the standard of care for LARC patients. Unfortunately, patients shows a wide range of response rates in which approximately 20% has a complete pathological response, whereas in 20 to 40% the response is poor or absent.

**Results:**

The following specific gene signature, able to discriminate responders' patients from non-responders, were founded: AKR1C3, CXCL11, CXCL10, IDO1, CXCL9, MMP12 and HLA-DRA. These genes are mainly involved in immune system pathways and interact with drugs traditionally used in the adjuvant treatment of rectal cancer.

**Discussion:**

The present study suggests that new ideas for therapy could be found not only limited to studying genes differentially expressed between the two groups of patients but deepening the mechanisms, associated to response, in which they are involved.

**Methods:**

Gene expression studies performed by: Agostini *et al*., Rimkus *et al*. and Kim *et al*. have been merged through a meta-analysis of the raw data. Gene expression data-sets have been processed using A-MADMAN. Common differentially expressed gene (DEG) were identified through SAM analysis. To further characterize the identified DEG we deeply investigated its biological role using an integrative computational biology approach.

## INTRODUCTION

Colorectal cancer (CRC) is the third most common cancer in the world. According to recent epidemiological data, in 2014 nearly 50,310 patients died from CRC with a substantial equality distributed in both sex [1]. One-third of CRC are represented by rectal cancer (RC) and 40% of this are locally advanced rectal cancer (LARC). Although the genetic screening has been proven to greatly reduce the mortality for CRC, up to now there is a deficiency of clinical tools for an effective and early individuations of neoplasia. Preoperative chemoradiotherapy (pCRT), presently the standard of care for LARC [2–6], shows unfortunately a wide range of response rates in which approximately 20% of patients have a complete pathological response, whereas in 20 to 40% of patients the response is poor or absent [7, 8]. In addition, an ineffective pCRT is time-consuming, expensive and increases the perioperative morbidity. The development of novel approaches predictors of tumor response to pCRT are critical in reducing mortality in LARC and in sparing poorly responding patients from unnecessary treatments. Several studies have been performed to evaluate potential predictors of response after pCRT in rectal cancer, however the data are still unclear and controversial [9, 10]. Discrepancies among studies were mainly related to patient selection, sample size, study design, treatments and parameters used for tumor response evaluation. As a consequence, the only accepted marker to monitor colorectal cancer treatment, progression and disease relapse is the carcinoembryonic antigen (CEA), but its sensitivity and specificity, especially for early stage colorectal cancer, seems to be insufficient [10, 11]. Drug sensitivity in chemotherapy is thought to be attributable to the variations in the genetic background of cancer cells and gene expression signatures have a great potential for predicting therapeutic outcomes better than conventional clinical and pathological approaches [12]. In this landscape, gene signatures, based on rectal cancer cell expression profiling obtained by microarray technology, could be useful predictors of tumour response after pCRT. Some studies focusing on the prediction of response to treatment based on mRNA gene expression in LARC patients have been performed, but no overlap was found in the predictive genes list [12, 13] since the analyzed patients had received heterogeneous treatments.

For instance, in the paper from Watanabe *et al*. [14] patients were treated with radiotherapy alone, whereas Cetuximab was added to the conventional pCRT by Daemen *et al*. [15] and Debucquoy *et al*. [16]. In other studies [17–19] and a paper from our laboratory [20] the major limitation is probably the number of patients tested.

The “meta-analysis” of data produced by different but comparable studies could in theory increase the statistical power and reliability of the study beyond the original experimental design. On the other hand, different datasets are often produced using different platforms, presenting some challenges for meaningful data integration [21]. In this scenario there are two main possible strategies to integrate the results of independent gene expression studies: *i)* the combination of retrospective analysis results (i.e. final lists of differentially expressed genes); *ii) de-novo* analysis (re-analysis) of raw data [22, 23].

In this study, we performed a *de novo* meta-analysis on data from rectal tumor tissue to identify a gene set associated to pCRT response in association with the disease free survival data. The Annotation-based Microarray Data Meta-ANalysis tool (A-MADMAN; http://compgen.bio.unipd.it/bioinfo/amadman/) [24], an open source web application that was already successfully used in several studies [25–27] was used to retrieve, annotate and integrate gene expression data from three independent datasets.

In addition, we applied an integrated computational approach to interpolate data derived from genetic signatures and proteomic analyses to achieve more reliable predictions in the response to treatment.

## RESULTS

### Responders *vs* non-responders: meta-analysis of clinical and gene expression data

Initially, we focused on clinical data to understand whether there was a correlation between responses to therapy with the overall survival. Then, we integrated gene expression signatures with both functional information, such as: biological processes (BP), cellular components (CC) and molecular functions (MF); and clinical data to search for differences in gene expression linked to pCRT response and to obtain information on the molecular mechanisms that could be involved in differential survival.

The array-based gene expression profiling in LARC tumor samples in relation to pCRT response of patients has been evaluated in a cohort of 85 samples, coming from the three independent studies. Data have been collected and integrated to conduct a meta-analysis aiming at the identification of a robust gene signature able to predict chemo-radio resistance.

In this regard, we compared the gene lists identified by the three independent studies. No shared markers resulted after data intersection, including XRCC3 the promising gene identified in our study, probably due to the different data analysis procedures (Supplemental Table 1). To address this issue, we processed together the raw data retrieved by the three studies to obtain a larger statistical sample. Through a *de-novo* integration of gene expression data in a single matrix we selected 277 genes with an expression profiles significantly deregulated, while low expression variation have been filtered out using the interquartile range (IQR) criteria (Supplemental Table 2).

### A signature of chemoradio resistance in LARC

SAM analysis was performed on 277 significantly expressed genes between responders (R) and non-responders (NR) groups. Through this method, we found a specific gene signature able to discriminate R from NR: one gene was significantly under-expressed (AKR1C3 [Gene ID: 8644]), while six genes were over-expressed (CXCL11[Gene ID: 6373], CXCL10[Gene ID: 3627], IDO1[Gene ID: 3620], CXCL9[Gene ID: 4283], MMP12[Gene ID: 4321] and HLA-DRA[Gene ID: 3122]) in responders group with FDR < 1% and *q*-value = 0 (Table 1). The SAM plot with significantly up- and down-regulated genes, the heatmap of Differentially Expressed Genes (DEG) expression profiles and the boxplot showing expression variation of DEGs in considered groups are reported in (Supplemental Figure 1).

**Table 1:** SAM results, genes deregulated in Responders (R) group

Probe ID	Gene Symbol	Expression	meanNR	mean R	Description
GC10P005126_at	AKR1C3Gene ID: 8644	↓R	8.818	7.766	Encodes a member of the aldo/keto reductase superfamily. It catalyzes the conversion of aldehydes and ketones to alcohols.
GC04M077145_at	CXCL11 Gene ID: 6373	↑R	3.787	4.875	Is a CXC member of the chemokine superfamily. Chemotactic for interleukin-activated T-cells but not unstimulated T-cells, neutrophils or monocytes, ligand for the receptor CXCR3.
GC04M077132_at	CXCL10 Gene ID: 3627	↑R	6.693	7.680	Encodes a chemokine of the CXC subfamily and ligand for the receptor CXCR3.
GC08P039891_at	IDO1Gene ID: 3620	↑R	6.095	7.007	Encodes a heme enzyme that catalyzes the first and rate-limiting step in tryptophan catabolism to N-formyl-kynurenine, immune-modulatory properties.
GC04M077112_at	CXCL9 Gene ID: 4283	↑R	6.130	7.064	The function has not been specifically defined; however, it is thought to be involved in T cell trafficking, ligand for the receptor CXCR3
GC11M102238_at	MMP12 Gene ID: 4321	↑R	8.819	9.580	Encodes a protein of the matrix metalloproteinase (MMP) involved in the breakdown of extracellular matrix.
GC06P032434_at	HLA-DRA Gene ID: 3122	↑R	7.759	8.430	Encodes a protein that binds peptides derived from antigens that access the endocytic route of antigen presenting cells (APC) and presents them on the cell surface for recognition by the CD4 T-cells.

Only genes with FDR < 1% and *q*-value = 0 are listed

**Figure 1:**
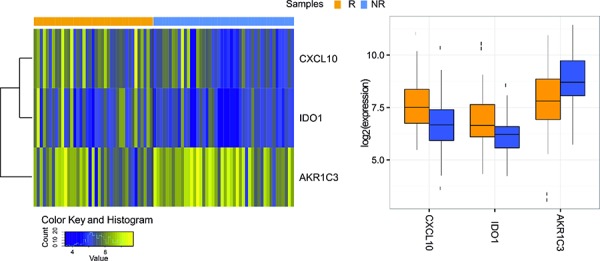
Three genes were validated using Multtest package (Benjamini–Hochberg procedure with *p* = 0.05 assumption) As shown by the heatmap of gene expression profiles in considered samples (left) and by the Boxplot (right), IDO1 and CXCL10 genes are up-regulated in Responders group, while AKR1C3 is down-regulated.

Gene ontology (GO) analysis has been performed on the seven selected genes. DAVID web-based tool was used to investigate biological processes (BP), cellular components (CC) and molecular functions (MF) shared by them. Ordered by statistical significance, BP results showed that five out of seven genes (CXCL10, CXCL11, CXCL9, IDO1, HLA-DRA) were involved in defense response and four genes (CXCL10, CXCL11, CXCL9, IDO1) in inflammatory response pathway. Four genes (CXCL10, CXCL11, CXCL9, MMP12) encoded for proteins commonly located in the extra-cellular regions, according to CC information. Three genes (CXCL10, CXCL11, CXCL9) showed the same MF sharing chemokine, cytokine activity and chemokine receptor binding. The most significant three out of seven SAM genes (AKR1C3, CXCL10 and IDO1, Figure 1) were further validated by the *multtest* Benjamini–Hochberg procedure with *p* = 0.05 assumption.

We also estimated the prediction error via cross-validation using PAM machine learning method, which provides a list of significant genes able to discriminate between responders and non-responders.

No genes were found (PAM, Supplemental Figure 2) to be predictive in distinguishing responders from non-responders groups. A comprehensive summary (Table 2) was created to summarize the analyses results.

**Table 2:** Summary table for statistical tests performed to find differentially expressed genes between responders and non-responders groups on 85 samples

Gene	Expression	SAM	PAM	Multtest	ST - lFDR	ST - qval
AKR1C3	↓R	passed	failed	passed	failed	failed
CXCL11	↑R	passed	failed	failed	failed	failed
CXCL10	↑R	passed	failed	passed	failed	failed
IDO1	↑R	passed	failed	passed	failed	failed
CXCL9	↑R	passed	failed	failed	failed	failed
MMP12	↑R	passed	failed	failed	failed	failed
HLA-DRA	↑R	passed	failed	failed	failed	failed

To further strengthen our results, we validated the predictive power of genetic signature by an elaboration of data from two independent TCGA CRC cohorts, respectively (*n* = 237) and (*n* = 881); in two out seven DEG, one over-expressed and one under-expressed. This result, in complete accordance with our data, showed an over-expression of IDO1 and an under-expression of AKR1C3 in responders patients (Supplemental figure 3) according to Residual tumor classification (see Methods paragraph), confirming the same trend obtained in our study [28].

### Multivariate analysis

We have considered a multivariate linear model to relate AKR1C3, CXCL10 and IDO1 to the variables with 100% of values available for each paper (Batch, Age, Sex, pT, LN, Metastases and Response). Multiplicity corrections have been performed using Holm-Bonferroni method to control the family wise error rate. A test was deemed significant if the corresponding adjusted *p*-value was below 0.05. We found that AKR1C3 showed a statistical significant decrease if the observations belong to pT(1) (estimate −1.928e+09, corrected *p* = 0.029440572) and a significant increase if the observations belong to Response(2) (estimate 1.309e+09, corrected *p* = 0.006074293).

### Genes correlated with survival

We further evaluated the entire gene expression profiles for two out of three studies to identify genes related to survival (Agostini *et al*.; Rimkus *et al*.). Four and seven genes were found to be significantly associated with death event using respectively local false discovery rate (lFDR < 0.05) and *q*-value (qval < 0.05) methods (Supplemental Figures 4a and 4b). Over-expression of INPP1[Gene ID: 3628], CYB5D1[Gene ID: 124637], KDELR3[Gene ID: 11015] and down-regulation of SLC26A2[Gene ID: 1836], VPS13C[Gene ID: 54832], PRKCQ[Gene ID: 5588] and TRIM2[Gene ID: 23321] genes characterized the event-related group.

### From genes and response mechanisms to new ideas for therapy?

**Figure 2:**
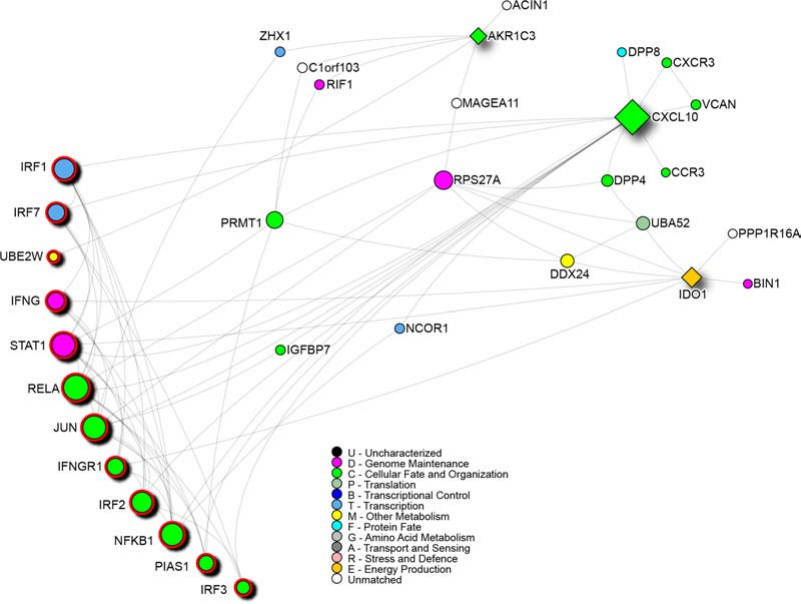
Network analysis of the three genes response-related signature positive to the *multtest* Squares indicate the signature genes, red outline highlights genes involved in the immune system pathway. Node colors are related to Gene Ontology terms as shown in the legend.

We utilized a systems biology approach to gain at least preliminary hypotheses of the mechanisms that could be involved in differential patients' response to therapy. The network analysis performed on AKR1C3, CXCL10 and IDO1 showed that these three genes are not directly connected but participate in a network of 33 genes with 80 interactions (Figure 2). The pathway analysis of the entire network reveals that the immune system pathway is enriched (*p* = 3.64E-07) and involves the most part of the central nodes in the network connecting the three genes (Supplememtal table 3).

**Figure 3:**
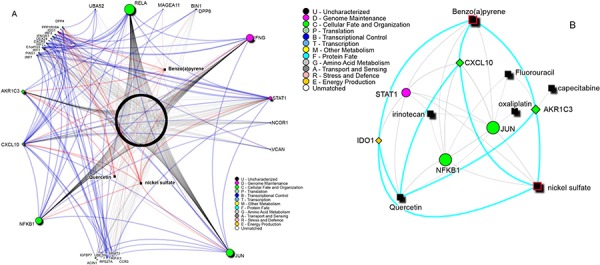
A. Known chemical compounds targeting the signature network reported in Figure 2 Labeled compounds are the only ones targeting all three-signature proteins. Blue edges show protein-protein interactions, red edges connections between the labeled compounds and their direct targets. **B.** Drugs network summary. In black are reported drugs and chemical compounds. The drugs used in rectal cancer treatment and their targets are shown. Highlighted in red are compounds that are known to be related to CRC carcinogenesis. Light gray edges show interactions between signature genes and drugs targeting all of them.

We then used “The Comparative Toxicogenomics Database” CTD, to highlight which known chemical compound targets the genes of the previously built network (Figure 3, panel a). Benzo(a)pyrene, quercetin and nickel sulfate are the only ones targeting all three signature genes. Drugs traditionally used in the adjuvant treatment of rectal cancer were reported in the network dependencies: capecitabine, 5-fluorouracil (both targeting AKR1C3), irinotecan and oxaliplatin (Figure 3 panel b) and (Supplemental table 4).

**Figure 4:**
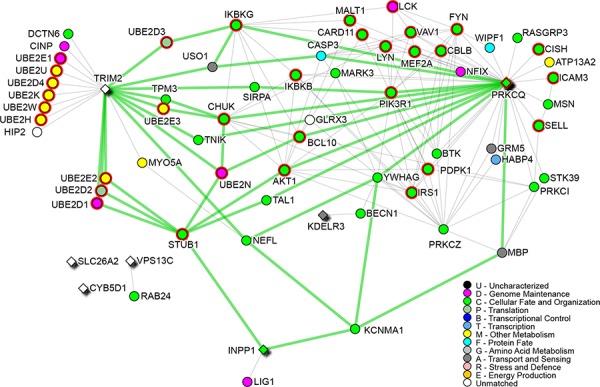
Survival signature network Survival genes are diamond-shape nodes, while highlighted nodes are part of the Immune system pathway. Node colors are related to Gene Ontology terms as shown in the legend. Green edges are shortest paths between survival genes.

The network analysis of the survival genes has also been performed. A network of 69 nodes with 157 edges showed that the immune system pathway as the most enriched one (*p* value = 6.13E-22) (Figure 4).

## DISCUSSION

Clinically, preoperative chemoradiotherapy, is the worldwide standard regime for locally advanced rectal cancer. However, the therapy outcome is very heterogeneous, and ineffective therapy is time-consuming, economically ineffective, and leads to increased morbidity. Therefore, reliable prediction of therapy response by novel approaches is urgently awaited. In the past, gene expression profiling has been used to define biomarkers for tumor response to pCRT by several groups. However, single-center studies rely on relatively low case numbers, leading to very limited levels of overlap between the findings, as our own results demonstrate for three recent studies (Supplemental Table 1). Therefore, we have chosen here a new approach based on de novo bioinformatics analysis of the original data, a powerful strategy for the generation of new potential biomarkers for therapy response stratification.

Firstly, an interesting things approaching to this manuscript is that none of our predictive genes are consistent with other comparable studies before mentioned. This apparent discrepancy in results, is mainly due to the high heterogeneity in a series of parameters including: number of patients enrolled, studies design and methods, primary end-point, treatment's protocol, criteria used for evaluation of treatment response, microarray platform and statistic tools. For example, Debucquoy *et al*. while using our similar approach, treat LARC patients with a primary end-point of evaluating the efficacy of a COX-2 inhibitor combined with canonical chemoradiotherapy. Instead, Ghadimi *et al*. used both a different therapeutic treatment protocol and responders/non-responders evaluation method with our same primary scope. Conversely, Watanabe *et al*. and Brettingham-Moore *et al*. studies is the most comparable works in term of study design and practical approach. Watanabe *et al*. enrolled fifty-two rectal patients who underwent pCRT and through microarray genes expression profile identified a signature of 33 differently expressed genes discriminating responders from non-responders patients. The list of discriminating genes included growth factor, apoptosis, cell proliferation, signal transduction, or cell adhesion–related genes but none are in common with our findings. Brettingham-Moore *et al*. highlight how the result obtained with microarray approach fail to reach a sufficient sensitivity and specificity in stratifying responders from non-responders patients, with a poor predictive power, reproducibility and a high rate of false-positive. However, using an alternative computational approach they found that a particular biological pathway was principally involved and high correlated to patient's response to treatment: the TNF/NF-*k*B pathway. Consistently with our result they found that inflammation, especially which involved in tumor microenvironment, often cooperate with a complex network of signals determining sensitivity to chemoradiotherapy.

Secondly, the results here presented support the growing evidence of tumor microenvironment (TME) involvement in neoplastic progression [29]. While some aspects of TME are now well characterized such as tumor angiogenesis sustaining and reshaping of extracellular matrix, the impact of TME on tumor grown, progression and response to therapy is still not fully clarified. In this landscape, the intersection between tumor and immune system is increasingly taking on the characteristics of a double-edge sword. In particular the Infiltrating Immune Cells (IIC) seems to, directly and indirectly, supply a series of mediators able to sustain tumor unchecked proliferation and to interfere with prognosis and treatment response.

In the present *de novo* meta-analysis study, we found three genes: *CXCL10*, *IDO1* and *AKR1C3* associated to chemo-resistance in rectal cancer patients. These genes showed a common involvement in the immune system pathway by performing network analysis. The same result was obtained for the genes associated to survival. The adaptive immune system has been described to be involved in colorectal carcinogenesis and it has been already correlated to survival [30].

Of note, the three chemokines CXCL11, CXCL10, and CXCL9 were all found to be upregulated in therapy responders. These interferon-regulated CXC-family cytokines all bind to the same receptor, CXCR3[Gene ID: 2833], which is expressed on T_H1_-type T-cells and endothelial cells [31]. These cytokines, most prominently CXCL10 (IP-10), are involved in chemotaxis of monocyte and anti-tumoral T_H1_-type T-lymphocytes, in the regulation of cell growth and have been described to inhibit angiostasis. For cell-mediated immunity, CXCL10 exerts a powerful thymus-dependent antitumor effect [32] and several studies have established a prominent role for NK cells in CXCL10-mediated antitumor activity [33].

Therefore, a deregulation of chemokine modulation in leukocyte recruitment might favour cancer promotion. For example, impaired production of CXCL10 might be the basis of decreased immune responses at sites of chronic inflammation that lead to neoplastic transformation [34] and more aggressive phenotypes. Low expression of CXCL10 has been previously associated with survival in stage II and III colorectal cancers and it could be used as a marker to better characterize high risk patients and maximize the benefits of adjuvant therapy [35]. Moreover, high CXCL10 mRNA expression is associated with a better tumor response to pCRT in LARC patients and may predict the outcome of pCRT in this malignancy [36]. It is noteworthy that the immune system was described as the main player in more than one part of this study. It is now well known that a chronically inflamed microenvironment, immune escape and immunosuppression are mechanisms intertwined with cancer development, to the point of being recognized as “immune hallmarks of cancer” [37]. Our results highlight the importance of a correct immune response in rectal cancer, and confirm the role of immune related pathways in modeling different responses to the same treatment [38]. Consistently with our results, the study of Zumwalt et al. [39] revealed that transcriptional expression and secretion of CXCR3 and CCR5, cognate chemokines, correlate with CD8+ T-cell infiltration and prolonged survival in colorectal cancer. Similarly, Sconocchia et al. [40] assessed that NK cell and CD8+ T cell crosstalk in the tumor microenvironment may benefit patient outcome and further, that the enumeration of infiltrating NK and CD8+ T cells in CRC tumors may provide useful prognostic information.

The importance of CXCL10 and CXCL9 in the generation of an efficient antitumor immune response is further supported by their prominent role as mediators of the antitumor effect of IL-12[Gene ID: 3593], which is known as a potent immunoregulatory cytokine mediating tumor regression in a variety of tumor models [33, 41].

IDO1 is a fundamental enzyme in the tryptophan catabolism pathway and the data available in literature appear conflicting. A study on IDO1 expression in primary colorectal tumours demonstrated that higher IDO1 expression at the tumour invasion front correlates with progressive disease and impaired clinical outcome. At stage-III colorectal cancer patients, decisions for adjuvant treatment are weighed against possible side effects in patients with important comorbidity and the analysis of IDO1 expression at the tumour invasion front was suggested to be of additional value in the decision process [42]. Moreover, IDO1 has been demonstrated to promote colitis-associated tumorigenesis in mice, independent of its ability to limit T-cell–mediated immune surveillance. In the mechanism proposed, IDO1 regulates the suppression of tumor-reactive effector T cells and promotes the regulation of T cell activation limiting tumor immune-surveillance and thus facilitating tumor progression. Concurrently, it stimulates proliferation in the neoplastic epithelium through activated nuclear b-catenin [43].

AKR1C3 is an aldo–keto reductase that has been described as responsible for gaining resistance against cisplatin treatment when overexpressed in colon cancer cells together with AKR1C1[Gene ID: 1645] [44].

AKR1 family genes (C1 and C3) are potential markers for cisplatin resistance of colon cancer cells. In response to oxidative stress including ROS and electrophiles, Nrf2 is released from the Nrf2–Kelch-like ECH-associated protein 1 (Keap1 [Gene ID: 9817]) complex by modification of Cys in Keap1. Nrf2 translocates into the nucleus to binds antioxidant response elements, inducing a variety of phase II enzymes involved in drug metabolism, such as NAD(P)H quinone oxidoreductase-1, heme oxygenase-1 and the AKRs (1C1, 1C2, 1C3 and 1B10) [45, 46].

Since cisplatin induces oxidative stress, it has been suggested that the over-expression of AKR1C1 and AKR1C3 in cisplatin resistance cells results induced from Nrf2 activation through continuous formation of ROS and HNE. The positive relationship between the up-regulation of the two AKRs and the development of cisplatin resistance, seems to indicate that high expression of AKR1 genes (C1 and C3) is a crucial factor in the ineffectiveness of cisplatin therapy for colon cancer.

Drugs used in the adjuvant treatment of rectal cancer (capecitabine, fluorouracil, irinotecan and oxaliplatin) are present in the network connecting the three genes (Figure 3, panel b).

A search for compounds that target all three genes pointed at benzo(a)pyrene, nickel sulfate and quercetine. Benzo(a)pyrene has been linked to colorectal cancer development [47] while nickel sulfate is a known carcinogen [48]. On the contrary quercetin has been associated to colorectal cancer prevention and treatment of rectal and ileal adenomas [49, 50]. A further analysis on the interactions revealed that the two carcinogens induce the expression of the three genes while quercetin induces the expression of IDO1 but inhibits the expression of CXCL10 and AKR1C3. As IDO1 is up-regulated and AKR1C3 is down-regulated in responders, quercetin might be a good candidate in support to adjuvant chemotherapy in non-responders patients.

Interestingly, STAT1[Gene ID: 6772], NFKB1[Gene ID: 4790] and JUN[Gene ID: 3725] genes, three crucial signaling molecules in cancer cells, have been further identified as common targets of the three compounds. The enrichment of the immune system pathway was confirmed also considering the six genes targeted by the three compounds (*p* = 0.0228). Notably, STAT1, NFKB1 and JUN are also targeted by irinotecan while fluorouracil targets NFKB1 and JUN and oxaliplatin targets JUN.

The present study furthermore, allowed the identification of a subclass of genes related to survival. Among these genes, INPP1 in particular stands out for which its overexpression is positively correlated to death-event. The over expression in colorectal cancer of INPP1 was primary described by Bustin and colleagues [51]. This gene produces an important enzyme involved in the Inositol signaling pathway that play a crucial role in cellular proliferation, differentiation and apoptosis [52, 53]. INPP1 belongs to a class of hydrolases acting specifically on position 1 phosphate of both InsP2 and InsP3. The production derived from its catalysis functions as effector molecule in favor of DNA Polymerase α, required for the priming of Okazaki fragments during elongation [54], increasing its fidelity and activity of replication [55]. A deregulated activity of INPP1 has also been demonstrated in colorectal cancer and in prostate cancer, where it has been proposed as therapeutic target [56]. In concert with our data, we speculate that an over-activation of INPP1, besides increasing the activity and stability of DNA Polymerase α promotes cancer cell replication, stimulates the generation of some second messengers such as diacylglycerol and InsP3 which are important in the activation of protein kinase C, responsible for survival and propagation of proliferation-enhancing stimuli.

Another noteworthy gene is CYB5D1, which encodes for a protein belonging to the family of Hpr6, which was found to increase the resistance of tumor cells to DNA damaging agents [57]. This study is the first, up to now, that positively correlates overexpression of CYB5D1 with an increased patients risk. In fact, CYB5D1 was already decribed in the study of breast and other cancer but its expression levels negatively correlates with patients survival, suggesting an opposing effects, which may be tumor-type specific [58, 59].

With respect to down-regulated genes, SLC26A2 alias DTD or DTDS, is an interesting gene, which positively correlated with death-event. This gene encodes for a protein responsible of sulfate transport across membrane. In cancer cell and especially in colorectal cancer cell, we assist to an impaired sulfation that occurs during the course of malignant transformation of colonic epithelium. A study proposed by Kannagi and colleagues demonstrated that cultured colorectal cancer cells have a markedly reduced expression of DTDST and the down expression dramatically increased growth rate and proliferation of cancer cells [60].

In conclusion, our study paves the way for an original approach in order to facing the dramatic increase of high throughput generated data. In fact, we must consider that the increasing use of high throughput assays shifted research from hypothesis-driven exploration to data-driven hypothesis generation, in this scenario an effective tool as A-MADMAN algorithm allow a fast, comprehensive and reliable statistical method to analyze heterogeneous data, coming from different analytical platforms. All these arguments suggest that new ideas for therapy, as well as new tools for patient stratification according to the individual molecular genetic risk profile could be found studying genes and mechanisms associated to therapy response.

## MATERIALS AND METHODS

### Gene expression datasets selection

In this study, three different gene expression datasets of pCRT-treated patients with LARC have been investigated. A total of 131 patient have been included. Only data belonging to original samples with > 70% of tumor cells and a comparable pCRT treatment protocol were used in our meta-analysis. Rimkus *et al*. [19] used 43 biopsy specimens of LARC patients receiving a pCRT standardized protocol: 45 Gy, 5-fluorouracil - 250 mg/m2/continuously followed by surgical resection six weeks later.

Agostini *et al*. (in press [20]) used tumor tissue obtained from 42 patients with LARC. All patients received pCRT using the standardized protocol as follow: 45–50.4 Gy; 5-fluorouracil - 225 mg/m2/continuously. Surgical excision has been performed six weeks after pCRT. In the two original studies, patients showing tumor regression grades 1–2 and 3–4 of the Mandard classification [61] were classified as responders and non-responders, respectively.

Kim *et al*. [18] used tumor tissues obtained from 46 patients with LARC. All patients were treated with radiotherapy with 50.4 Gy, followed by surgical excision six weeks later. The response to chemoradiotherapy was evaluated according to Dworak's tumor regression grade: grades 1–2 were considered non-responders, while grades 3–4 were considered as responders [62]. The main information for each study has been reported (Table 3). All genes ID have been retrieved from NCBI Gene database

**Table 3:** Summary table comparing the three studies used for meta-analysis

Manuscript	Study group	Arrays	Survival	Platform
Rimkus *et al*., CGH 2008	Germany	15	Yes	HG-U133Plus2.0
Agostini *et al*., (in press)	Italy	40	Yes	HG-U133Plus2.0
Kim *et al*.,DCR 2007	Korea	30	No	HG-U133A

### Data retrieval, annotation, integration and quality control

We used A-MADMAN web application for retrieving, annotating, organizing and analysing gene expression datasets of meta-analysis, originating from Affymetrix data. Interestingly, A-MADMAN allows the integrative analysis of data obtained from different Affymetrix platforms through a custom workflow by minimizing inter-series and inter-studies variability biases. This is based on the Robust Multichip Average (RMA) method [63] implemented in the Bioconductor R package ‘affy’, using GeneAnnote-based custom CDF [64]. Oligonucleotides common to all platforms were selected and each array was transformed into a virtual chip containing only the common probes. Background correction was applied for each platform before the oligonucleotides selection, while the summarization and the quantile normalization by RMA were performed only on shared probes.

R *pamr* package (CRAN Comprehensive R Archive Network; http://cran.r-project.org) was applied after normalization to remove batch effects.

Three series of Affymetrix data were downloaded and a total of 131 patients were considered. The data were obtained from two different generations of Affymetrix gene chip technologies that were not directly comparable: Human Genome U133A was used by Kim *et al*., while Human Genome U133 plus2.0 GeneChip Array was used by Rimkus *et al*. and Agostini *et al*.

Raw data (*CEL files*) and related metadata were imported and clinical and pathological information (age, sex, ethnicity, primary tumor site, stage, tumor grade) were used to build a local database, as previously described [24]. Briefly, metadata were associated to each sample as descriptive labels (tags) to subset samples into homogenous groups.

A quality check of the expression data was applied using a customized version of *ArrayQualityMetrics* Bioconductor package in R statistics environment (BioConductor, http://www.bioconductor.org) to discard those samples that were marked as outliers in more than two Quality Control (QC) metrics. Specifically, two main Probe-Level Method-based quality statistics, such as: Normalized Unscaled Standard Error (NUSE) with Relative Log Expression (RLE), and a QC metric recommend by Affymetrix (QC stats) were used to evaluate the overall quality of signals in each array. MA-plot before and after RMA was performed to identify biases associated with specific intensity classes [65].

By the A-MADMAN web interface, all samples were meta-analysed to derive an integrated gene expression matrix.

### Gene Expression Profiling

Statistical analyses were performed using the open-source R System software [The R Project for Statistical Computing, ver.2.12.2, http://www.r-project.org], BioConductor package [ver.2.7] and dChip software, including 12,167 probe sets and 85 samples resulting from annotation, integration and QC procedures above described.

Genes with low expression variation across all samples have been discarded using interquartile range (IQR) as filtering criteria; as a result, subsequent analysis has been performed using a total of 277 filtered probe sets.

A significance analysis of microarray (SAM) has been performed to find differentially expressed genes between responders (R) and non-responders (NR) groups. Less than one false positive-rated gene was found using false discovery rate (FDR) < 1% and *q*-value = 0 as cutoffs.

Shrunken centroid algorithm was further performed using Tibshirani's class prediction analysis of microarrays (PAM). The ‘*multtest*’ package was used for multiplicity control as described in Pollard, K. S., Gilbert, H. N., Ge, Y., Taylor, S. & Dudoit, S. (2011), multtest:

Resampling-based multiple hypothesis testing.

The false discovery rate (FDR) has been calculated by the Benjamini–Hochberg procedure and statistical threshold for significance was set to *p* = 0.05.

The functional annotation of gene lists derived from the above described analysis was performed by the DAVID (Database for Annotation, Visualization and Integrated Discovery) [web-tool: http://david.abcc.ncifcrf.gov/].

Survival data, available for 55 samples in two out of three original studies (Table 1) were considered also. We investigated the two databases to identify survival-related genes using “*shrinkage t*” statistics and “*st*” R package.

### TCGA data set analysis

Data from TCGA pilot project established by the NCI and NHGR were explored (22nd June 2015) for AKR1C3 and IDO1 expression in the TCGA colorectal cancer series. Information about TCGA and the investigators and institutions that constitute the TCGA research network can be found at “http://cancergenome.nih.gov”. Oncomine algorithms were used for the statistical analysis of the differences in AKR1C3 and IDO1 mRNA expression [66]. TCGA data series were grouped by Residual tumor, a parameter that reflects the effects of treatment as described by Hermanek *et al*. [28]. Briefly, R0 were considered complete/good response while R1 and R2 bad/absent response.

### Network Analysis

The biological role of selected genes, which enable the discrimination of two groups, is better characterized through an in-depth analysis of the molecular pathways in which they are involved. In this regard, we utilized a series of tools in order to investigated different biological network at different level: *a)* protein-protein interactions (PPIs) and enrichment analysis; *b)* common microRNAs interactions; *c)* common interaction with drugs.

The selected genes were initially characterized by retrieving physical PPIs from “I2D database ve, creating a PPI network that was visualized and analyzed in “NAViGaTOR 2.3 [67]”. We further performed a functional annotation and enrichment analysis of the network proteins using the built in pathway plugin, a study of the microRNAs targeting the PPI network using “mirDIP 1.1” [68], and a study of the drugs targeting the same network using (CTD). Moreover, to prioritize microRNAs in the network, data from published studies on response to neoadjuvant chemoradiotherapy and microRNA signatures were collected [69–71].

## SUPPLEMENTARY FIGURES AND TABLES





## Abbreviations

CRC: Colorectal cancer
RC: Rectal cancer
LARC: Locally advanced rectal cancer
pCRT: preoperative chemoradiotherapy
CEA: Carcinoembryonic antigen
BP: Biological process
CC: Cellular components
MF: Molecular functions
DEG: Differentially Expressed Genes.

## References

[1] Siegel RL, Miller KD, Jemal A (2015). Cancer statistics, 2015. CA: a cancer journal for clinicians.

[2] Sauer R, Becker H, Hohenberger W, Rodel C, Wittekind C, Fietkau R, Martus P, Tschmelitsch J, Hager E, Hess CF, Karstens JH, Liersch T, Schmidberger H, Raab R (2004). Preoperative versus postoperative chemoradiotherapy for rectal cancer. The New England journal of medicine.

[3] Kapiteijn E, Marijnen CA, Nagtegaal ID, Putter H, Steup WH, Wiggers T, Rutten HJ, Pahlman L, Glimelius B, van Krieken JH, Leer JW, van de Velde CJ (2001). Preoperative radiotherapy combined with total mesorectal excision for resectable rectal cancer. The New England journal of medicine.

[4] Baize N, Gerard B, Bleiberg H, Caroli-Bosc F, Berthier F, Legendre H, Pector JC, Hendlisz A (2006). Long-term survival of patients downstaged by oxaliplatin and 5-fluorouracil combination followed by rescue surgery for unresectable colorectal liver metastases. Gastroenterologie clinique et biologique.

[5] Bosset JF, Collette L, Calais G, Mineur L, Maingon P, Radosevic-Jelic L, Daban A, Bardet E, Beny A, Ollier JC (2006). Chemotherapy with preoperative radiotherapy in rectal cancer. The New England journal of medicine.

[6] Roh MS, Colangelo LH, O'Connell MJ, Yothers G, Deutsch M, Allegra CJ, Kahlenberg MS, Baez-Diaz L, Ursiny CS, Petrelli NJ, Wolmark N (2009). Preoperative multimodality therapy improves disease-free survival in patients with carcinoma of the rectum: NSABP R-03. Journal of clinical oncology: official journal of the American Society of Clinical Oncology.

[7] Minsky BD, Cohen AM, Kemeny N, Enker WE, Kelsen DP, Reichman B, Saltz L, Sigurdson ER, Frankel J (1992). Enhancement of radiation-induced downstaging of rectal cancer by fluorouracil and high-dose leucovorin chemotherapy. Journal of clinical oncology: official journal of the American Society of Clinical Oncology.

[8] Mohiuddin M, Hayne M, Regine WF, Hanna N, Hagihara PF, McGrath P, Marks GM (2000). Prognostic significance of postchemoradiation stage following preoperative chemotherapy and radiation for advanced/recurrent rectal cancers. International journal of radiation oncology, biology, physics.

[9] Smith FM, Reynolds JV, Miller N, Stephens RB, Kennedy MJ (2006). Pathological and molecular predictors of the response of rectal cancer to neoadjuvant radiochemotherapy. European journal of surgical oncology: the journal of the European Society of Surgical Oncology and the British Association of Surgical Oncology.

[10] Kuremsky JG, Tepper JE, McLeod HL (2009). Biomarkers for response to neoadjuvant chemoradiation for rectal cancer. International journal of radiation oncology, biology, physics.

[11] Locker GY, Hamilton S, Harris J, Jessup JM, Kemeny N, Macdonald JS, Somerfield MR, Hayes DF, Bast RC (2006). ASCO 2006 update of recommendations for the use of tumor markers in gastrointestinal cancer. Journal of clinical oncology: official journal of the American Society of Clinical Oncology.

[12] Brettingham-Moore KH, Duong CP, Greenawalt DM, Heriot AG, Ellul J, Dow CA, Murray WK, Hicks RJ, Tjandra J, Chao M, Bui A, Joon DL, Thomas RJ, Phillips WA (2011). Pretreatment transcriptional profiling for predicting response to neoadjuvant chemoradiotherapy in rectal adenocarcinoma. Clinical cancer research: an official journal of the American Association for Cancer Research.

[13] Kitahara O, Furukawa Y, Tanaka T, Kihara C, Ono K, Yanagawa R, Nita ME, Takagi T, Nakamura Y, Tsunoda T (2001). Alterations of gene expression during colorectal carcinogenesis revealed by cDNA microarrays after laser-capture microdissection of tumor tissues and normal epithelia. Cancer research.

[14] Watanabe T, Komuro Y, Kiyomatsu T, Kanazawa T, Kazama Y, Tanaka J, Tanaka T, Yamamoto Y, Shirane M, Muto T, Nagawa H (2006). Prediction of sensitivity of rectal cancer cells in response to preoperative radiotherapy by DNA microarray analysis of gene expression profiles. Cancer research.

[15] Daemen A, Gevaert O, De Bie T, Debucquoy A, Machiels JP, De Moor B, Haustermans K (2008). Integrating microarray and proteomics data to predict the response on cetuximab in patients with rectal cancer. Pacific Symposium on Biocomputing Pacific Symposium on Biocomputing.

[16] Debucquoy A, Roels S, Goethals L, Libbrecht L, Van Cutsem E, Geboes K, Penninckx F, D'Hoore A, McBride WH, Haustermans K (2009). Double blind randomized phase II study with radiation+5-fluorouracil+/−celecoxib for resectable rectal cancer. Radiotherapy and oncology: journal of the European Society for Therapeutic Radiology and Oncology.

[17] Ghadimi BM, Grade M, Difilippantonio MJ, Varma S, Simon R, Montagna C, Fuzesi L, Langer C, Becker H, Liersch T, Ried T (2005). Effectiveness of gene expression profiling for response prediction of rectal adenocarcinomas to preoperative chemoradiotherapy. Journal of clinical oncology: official journal of the American Society of Clinical Oncology.

[18] Kim IJ, Lim SB, Kang HC, Chang HJ, Ahn SA, Park HW, Jang SG, Park JH, Kim DY, Jung KH, Choi HS, Jeong SY, Sohn DK, Kim DW, Park JG (2007). Microarray gene expression profiling for predicting complete response to preoperative chemoradiotherapy in patients with advanced rectal cancer. Diseases of the colon and rectum.

[19] Rimkus C, Friederichs J, Boulesteix AL, Theisen J, Mages J, Becker K, Nekarda H, Rosenberg R, Janssen KP, Siewert JR (2008). Microarray-based prediction of tumor response to neoadjuvant radiochemotherapy of patients with locally advanced rectal cancer. Clinical gastroenterology and hepatology: the official clinical practice journal of the American Gastroenterological Association.

[20] Agostini M, Zangrando A, Pastrello C, D'Angelo E, Romano G, Giovannoni R, Giordan M, Maretto I, Bedin C, Zanon C, Digito M, Esposito G, Mescoli C, Lavitrano M, Rizzolio F, Jurisica I (2015). A functional biological network centered on XRCC3: a new possible marker of chemoradiotherapy resistance in rectal cancer patients. Cancer Biol Ther.

[21] Shibayama M, Maak M, Nitsche U, Gotoh K, Rosenberg R, Janssen KP (2011). Prediction of metastasis and recurrence in colorectal cancer based on gene expression analysis: ready for the clinic?. Cancers.

[22] Rung J, Brazma A (2013). Reuse of public genome-wide gene expression data. Nature reviews Genetics.

[23] Tseng GC, Ghosh D, Feingold E (2012). Comprehensive literature review and statistical considerations for microarray meta-analysis. Nucleic acids research.

[24] Bisognin A, Coppe A, Ferrari F, Risso D, Romualdi C, Bicciato S, Bortoluzzi S (2009). A-MADMAN: annotation-based microarray data meta-analysis tool. BMC bioinformatics.

[25] Sorani MD, Ortmann WA, Bierwagen EP, Behrens TW (2010). Clinical and biological data integration for biomarker discovery. Drug discovery today.

[26] Dawany NB, Tozeren A (2010). Asymmetric microarray data produces gene lists highly predictive of research literature on multiple cancer types. BMC bioinformatics.

[27] Jupiter D, Chen H, VanBuren V (2009). STARNET 2: a web-based tool for accelerating discovery of gene regulatory networks using microarray co-expression data. BMC bioinformatics.

[28] Hermanek P, Wittekind C (1994). Residual tumor (R) classification and prognosis. Seminars in surgical oncology.

[29] Hanahan D, Coussens LM (2012). Accessories to the crime: functions of cells recruited to the tumor microenvironment. Cancer cell.

[30] Mlecnik B, Tosolini M, Charoentong P, Kirilovsky A, Bindea G, Berger A, Camus M, Gillard M, Bruneval P, Fridman WH, Pages F, Trajanoski Z, Galon J (2010). Biomolecular network reconstruction identifies T-cell homing factors associated with survival in colorectal cancer. Gastroenterology.

[31] Groom JR, Luster AD (2011). CXCR3 ligands: redundant, collaborative and antagonistic functions. Immunology and cell biology.

[32] Luster AD, Leder P (1993). IP-10, a -C-X-C- chemokine, elicits a potent thymus-dependent antitumor response *in vivo*. The Journal of experimental medicine.

[33] Yao L, Sgadari C, Furuke K, Bloom ET, Teruya-Feldstein J, Tosato G (1999). Contribution of natural killer cells to inhibition of angiogenesis by interleukin-12. Blood.

[34] Tannenbaum CS, Tubbs R, Armstrong D, Finke JH, Bukowski RM, Hamilton TA (1998). The CXC chemokines IP-10 and Mig are necessary for IL-12-mediated regression of the mouse RENCA tumor. Journal of immunology.

[35] Jiang Z, Xu Y, Cai S (2010). CXCL10 expression and prognostic significance in stage II and III colorectal cancer. Molecular biology reports.

[36] Li C, Wang Z, Liu F, Zhu J, Yang L, Cai G, Zhang Z, Huang W, Cai S, Xu Y (2014). CXCL10 mRNA expression predicts response to neoadjuvant chemoradiotherapy in rectal cancer patients. Tumour biology: the journal of the International Society for Oncodevelopmental Biology and Medicine.

[37] Cavallo F, De Giovanni C, Nanni P, Forni G, Lollini PL (2011). 2011: the immune hallmarks of cancer. Cancer immunology, immunotherapy: CII.

[38] Grivennikov SI, Greten FR, Karin M (2010). Immunity, inflammation, and cancer. Cell.

[39] Zumwalt TJ, Arnold M, Goel A, Boland CR (2015). Active secretion of CXCL10 and CCL5 from colorectal cancer microenvironments associates with GranzymeB+ CD8+ T-cell infiltration. Oncotarget.

[40] Sconocchia G, Eppenberger S, Spagnoli GC, Tornillo L, Droeser R, Caratelli S, Ferrelli F, Coppola A, Arriga R, Lauro D, Iezzi G, Terracciano L, Ferrone S (2014). NK cells and T cells cooperate during the clinical course of colorectal cancer. Oncoimmunology.

[41] Kanegane C, Sgadari C, Kanegane H, Teruya-Feldstein J, Yao L, Gupta G, Farber JM, Liao F, Liu L, Tosato G (1998). Contribution of the CXC chemokines IP-10 and Mig to the antitumor effects of IL-12. Journal of leukocyte biology.

[42] Ferdinande L, Decaestecker C, Verset L, Mathieu A, Moles Lopez X, Negulescu AM, Van Maerken T, Salmon I, Cuvelier CA, Demetter P (2012). Clinicopathological significance of indoleamine 2,3-dioxygenase 1 expression in colorectal cancer. British journal of cancer.

[43] Thaker AI, Rao MS, Bishnupuri KS, Kerr TA, Foster L, Marinshaw JM, Newberry RD, Stenson WF, Ciorba MA (2013). IDO1 metabolites activate beta-catenin signaling to promote cancer cell proliferation and colon tumorigenesis in mice. Gastroenterology.

[44] Matsunaga T, Hojo A, Yamane Y, Endo S, El-Kabbani O, Hara A (2013). Pathophysiological roles of aldo-keto reductases (AKR1C1 and AKR1C3) in development of cisplatin resistance in human colon cancers. Chemico-biological interactions.

[45] MacLeod AK, McMahon M, Plummer SM, Higgins LG, Penning TM, Igarashi K, Hayes JD (2009). Characterization of the cancer chemopreventive NRF2-dependent gene battery in human keratinocytes: demonstration that the KEAP1-NRF2 pathway, and not the BACH1-NRF2 pathway, controls cytoprotection against electrophiles as well as redox-cycling compounds. Carcinogenesis.

[46] Klaassen CD, Reisman SA (2010). Nrf2 the rescue: effects of the antioxidative/electrophilic response on the liver. Toxicology and applied pharmacology.

[47] Sinha R, Kulldorff M, Gunter MJ, Strickland P, Rothman N (2005). Dietary benzo[a]pyrene intake and risk of colorectal adenoma. Cancer epidemiology, biomarkers and prevention: a publication of the American Association for Cancer Research, cosponsored by the American Society of Preventive Oncology.

[48] Salnikow K, Zhitkovich A (2008). Genetic and epigenetic mechanisms in metal carcinogenesis and cocarcinogenesis: nickel, arsenic, and chromium. Chemical research in toxicology.

[49] Kyle JA, Sharp L, Little J, Duthie GG, McNeill G (2010). Dietary flavonoid intake and colorectal cancer: a case-control study. The British journal of nutrition.

[50] Cruz-Correa M, Shoskes DA, Sanchez P, Zhao R, Hylind LM, Wexner SD, Giardiello FM (2006). Combination treatment with curcumin and quercetin of adenomas in familial adenomatous polyposis. Clinical gastroenterology and hepatology: the official clinical practice journal of the American Gastroenterological Association.

[51] Li SR, Gyselman VG, Lalude O, Dorudi S, Bustin SA (2000). Transcription of the inositol polyphosphate 1-phosphatase gene (INPP1) is upregulated in human colorectal cancer. Molecular carcinogenesis.

[52] Majerus PW (1992). Inositol phosphate biochemistry. Annual review of biochemistry.

[53] Berridge MJ, Irvine RF (1989). Inositol phosphates and cell signalling. Nature.

[54] Sylvia V, Curtin G, Norman J, Stec J, Busbee D (1988). Activation of a low specific activity form of DNA polymerase alpha by inositol-1,4-bisphosphate. Cell.

[55] Srivastava V, Tilley R, Miller S, Hart R, Busbee D (1992). Effects of aging and dietary restriction on DNA polymerases: gene expression, enzyme fidelity, and DNA excision repair. Experimental gerontology.

[56] Lee CH, Liu M, Sie KL, Lee MS (1996). Prostate-specific antigen promoter driven gene therapy targeting DNA polymerase-alpha and topoisomerase II alpha in prostate cancer. Anticancer research.

[57] Yasrebi H, Sperisen P, Praz V, Bucher P (2009). Can survival prediction be improved by merging gene expression data sets?. PloS one.

[58] Grade M, Hormann P, Becker S, Hummon AB, Wangsa D, Varma S, Simon R, Liersch T, Becker H, Difilippantonio MJ, Ghadimi BM, Ried T (2007). Gene expression profiling reveals a massive, aneuploidy-dependent transcriptional deregulation and distinct differences between lymph node-negative and lymph node-positive colon carcinomas. Cancer research.

[59] Craven RJ (2008). PGRMC1: a new biomarker for the estrogen receptor in breast cancer. Breast cancer research: BCR.

[60] Yusa A, Miyazaki K, Kimura N, Izawa M, Kannagi R (2010). Epigenetic silencing of the sulfate transporter gene DTDST induces sialyl Lewisx expression and accelerates proliferation of colon cancer cells. Cancer research.

[61] Mandard AM, Dalibard F, Mandard JC, Marnay J, Henry-Amar M, Petiot JF, Roussel A, Jacob JH, Segol P, Samama G (1994). Pathologic assessment of tumor regression after preoperative chemoradiotherapy of esophageal carcinoma. Clinicopathologic correlations. Cancer.

[62] Dworak O, Keilholz L, Hoffmann A (1997). Pathological features of rectal cancer after preoperative radiochemotherapy. International journal of colorectal disease.

[63] Irizarry RA, Hobbs B, Collin F, Beazer-Barclay YD, Antonellis KJ, Scherf U, Speed TP (2003). Exploration, normalization, and summaries of high density oligonucleotide array probe level data. Biostatistics.

[64] Ferrari F, Bortoluzzi S, Coppe A, Sirota A, Safran M, Shmoish M, Ferrari S, Lancet D, Danieli GA, Bicciato S (2007). Novel definition files for human GeneChips based on GeneAnnot. BMC bioinformatics.

[65] Heber S, Sick B (2006). Quality assessment of Affymetrix GeneChip data. Omics: a journal of integrative biology.

[66] http://www.oncomine.org/resource/login.html.

[67] Brown KR, Otasek D, Ali M, McGuffin MJ, Xie W, Devani B, Toch IL, Jurisica I (2009). NAViGaTOR: Network Analysis, Visualization and Graphing Toronto. Bioinformatics. (Oxford, England).

[68] Shirdel EA, Xie W, Mak TW, Jurisica I (2011). NAViGaTing the micronome—using multiple microRNA prediction databases to identify signalling pathway-associated microRNAs. PloS one.

[69] Drebber U, Lay M, Wedemeyer I, Vallbohmer D, Bollschweiler E, Brabender J, Monig SP, Holscher AH, Dienes HP, Odenthal M (2011). Altered levels of the onco-microRNA 21 and the tumor-supressor microRNAs 143 and 145 in advanced rectal cancer indicate successful neoadjuvant chemoradiotherapy. International journal of oncology.

[70] Della Vittoria Scarpati G, Falcetta F, Carlomagno C, Ubezio P, Marchini S, De Stefano A, Singh VK, D'Incalci M, De Placido S, Pepe S (2012). A specific miRNA signature correlates with complete pathological response to neoadjuvant chemoradiotherapy in locally advanced rectal cancer. International journal of radiation oncology, biology, physics.

[71] Garajova I, Svoboda M, Slaby O, Kocakova I, Fabian P, Kocak I, Vyzula R (2008). Possibilities of resistance prediction to neoadjuvant concomitant chemoradiotherapy in the treatment algorithm of patients with rectal carcinoma. Klinicka onkologie: casopis Ceske a Slovenske onkologicke spolecnosti.

