# Supplementary material for: An integrative approach for the identification of prognostic and predictive biomarkers in rectal cancer
Source: Oncotarget. 2015 Sep 2;6(32):32561–74. doi: 10.18632/oncotarget.4935 (PMC4741712; doi:10.18632/oncotarget.4935)
Supplement: Supplementary file 1 [file oncotarget-06-32561-s001.pdf]

SUPPLEMENTARY FIGURES AND TABLES

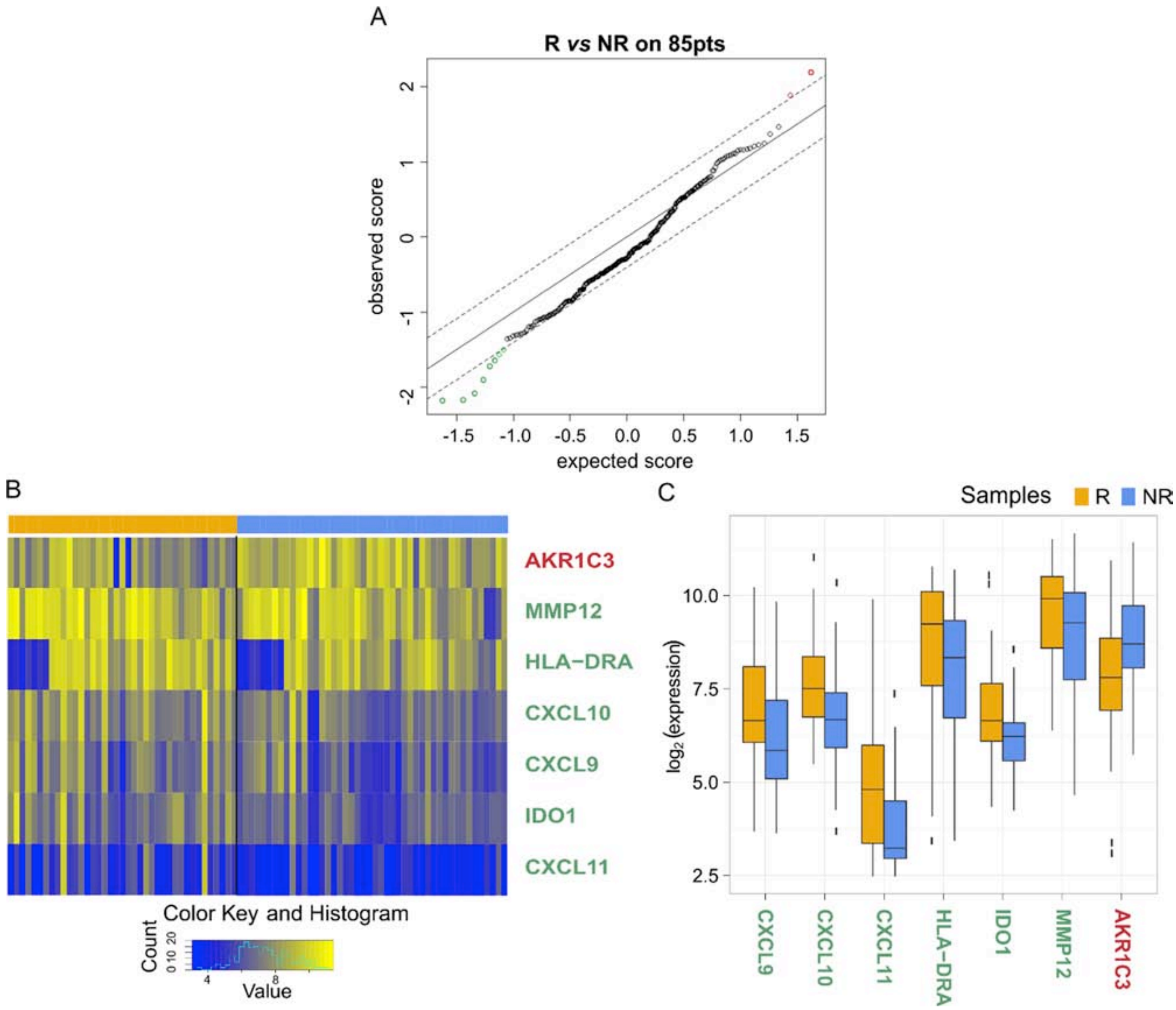

Supplementary Figure S1: Seven genes differentially expressed between responders and non-responders identified by SAM analysis. CXCL9, CXCL10, CXCL11, HLA-DRA, IDO1 and MMP12 were up-regulated while AKR1C3 was down-regulated in the responders group, and vice versa: **A.** SAM plot with significantly up-regulated genes labeled in red, down-regulated genes in green. Only genes beyond the 95% confidence interval have been considered; **B.** Heatmap of Differentially Expressed Genes (DEG) expression profiles in considered samples; **C.** Boxplot showing expression variation of DEGs in considered groups.

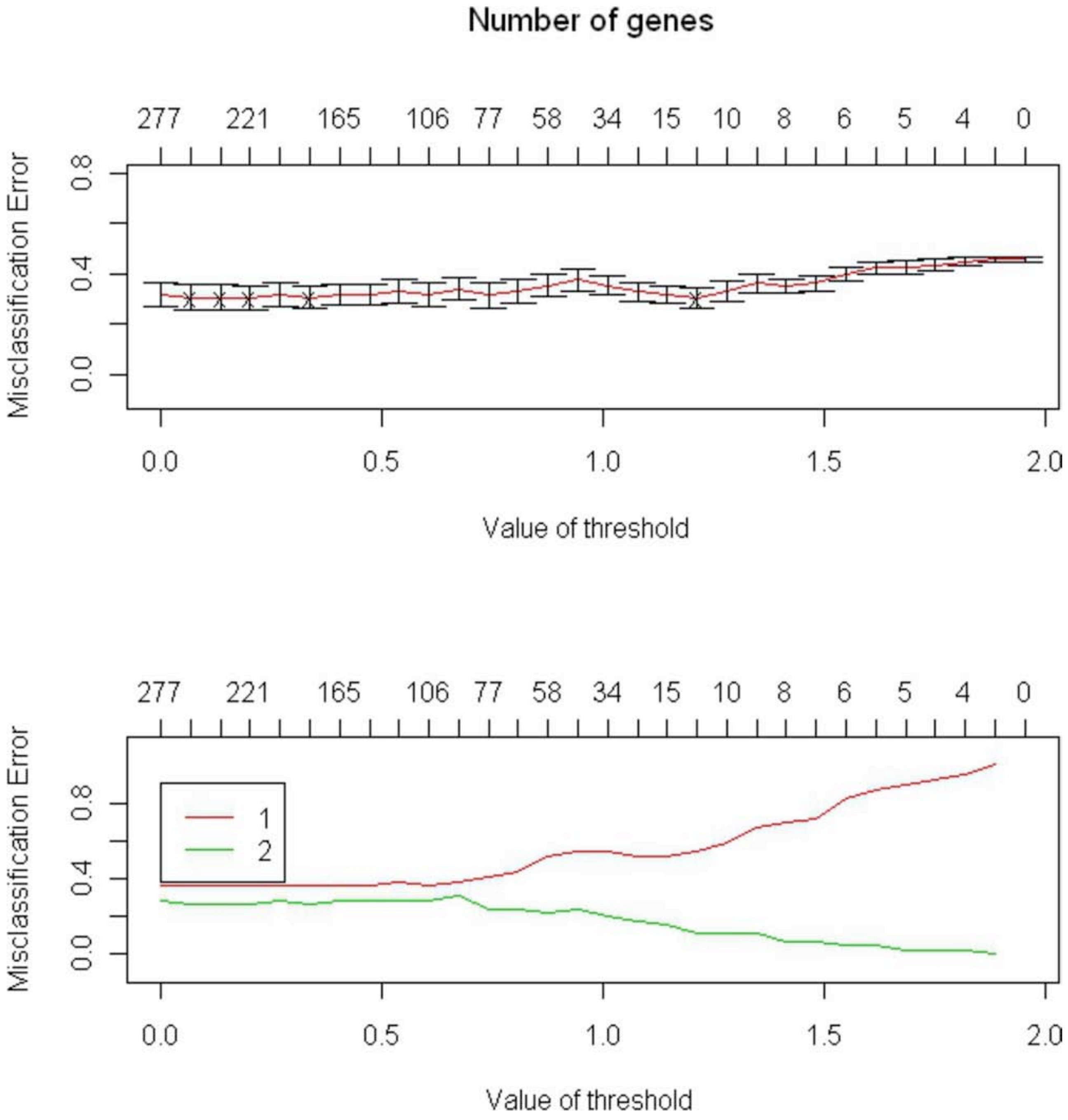

Supplementary Figure S2: PAM results: misclassification error rates between responders and non-responders using the set of 277 filtered genes.

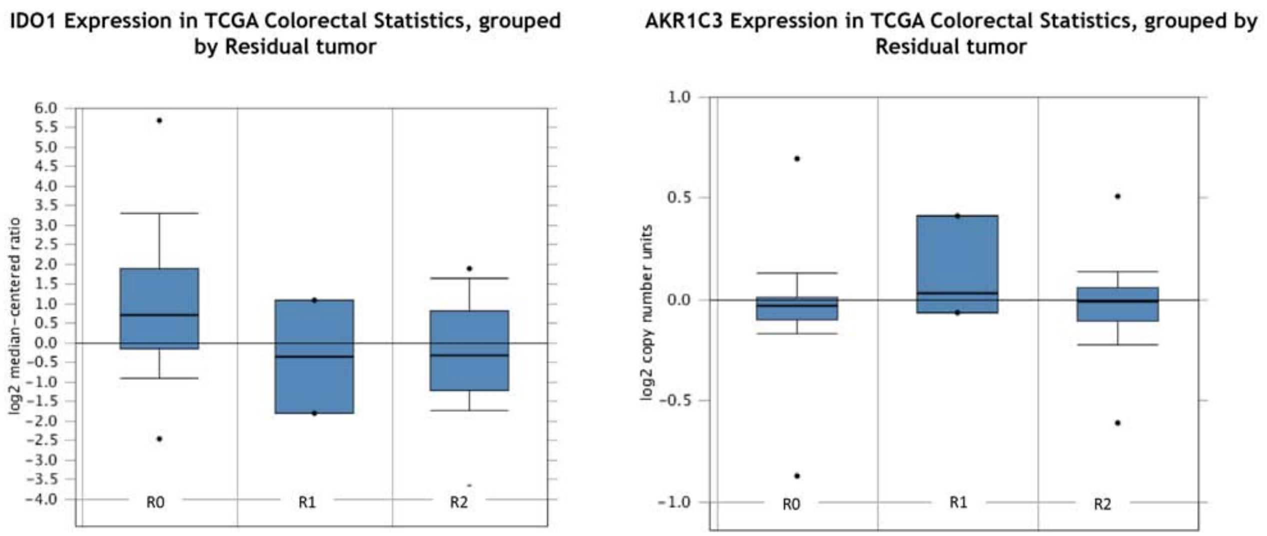

Supplementary Figure S3: TCGA data set analysis. IDO 1 and AKR1C3 expression level vs Residual tumor grade [R0=no residual tumor; R1=microscopic residual tumor; R2=macroscopic residual tumor;]. R0 were considered complete/good response while R1 and R2 bad/absent response.

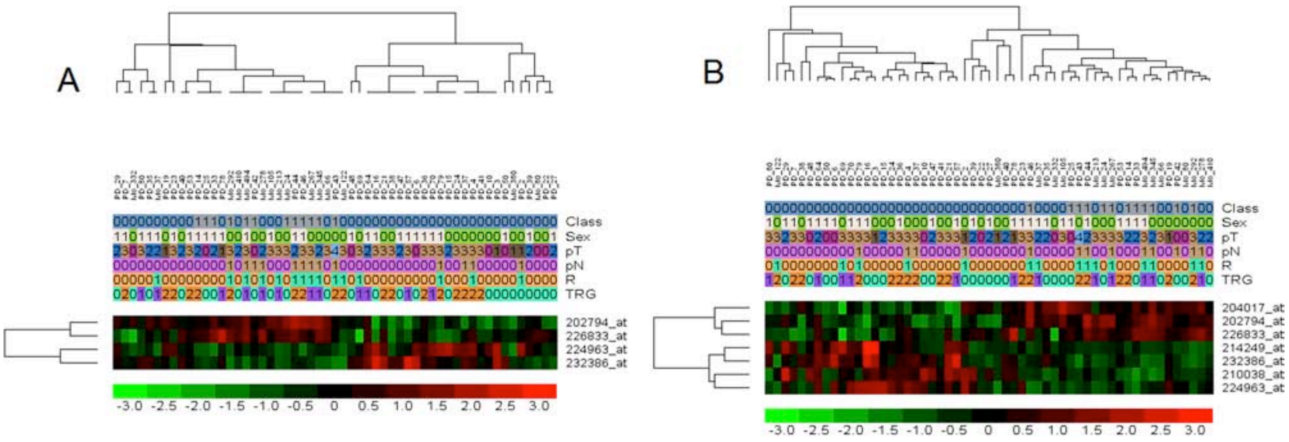

Supplementary Figure S4: A. Heatmap plot for genes related to survival. The analysis was performed using the four probe sets identified by local false discovery rate method. Probe ID: 202794_at=INPP1; 226833_at=CYB5D1; 224963_at=SLC26A2; 232386_at= VPS13C; **B.** The analysis was performed using the seven probe sets identified by *q*-value method. Class = 1 identifies the event. Probe ID: 204017_at=KDELR3; 202794_at=INPP1; 226833_at= CYB5D1; 214249_at=TRIM2; 232386_at=VPS13C; 210038_at=PRKCQ; 224963_at=SLC26A2. Abbreviations: pT=primary tumor; pN=primary node; R=residual tumor; TRG=tumor regression grade.

Supplementary Table S1: Differentially expressed genes between responders and non-responders groups identified by the three different studies

Kim *et al.*				Agostini *et al.*	Rimkus *et al.*	
AP2B1 Gene ID: 163	FLJ14054 Gene ID: 4883	LOC339692 Gene ID: 440895	RPS4Y1 Gene ID: 6192	XRCC3ID: 7517	ADPRHL2 Gene ID: 54936	PEX14 Gene ID: 5195
ARHGEF7 Gene ID: 8874	FLJ20674 Gene ID: 54621	LOC90379 Gene ID: 90379	SAE1 Gene ID: 10055	ZNF160ID: 90338	BLVRA Gene ID: 644	SDHC Gene ID: 6391
ASL Gene ID: 435	FLNA Gene ID: 2316	LOC92482 Gene ID: 92482	SEMA3C Gene ID: 10512	HFM1ID: 164045	C10orf4 Gene ID: 118924	SF3A1 Gene ID: 10291
BICD2 Gene ID: 23299	FOXO3A Gene ID: 2309	MAX Gene ID: 4149	SLC24A3 Gene ID: 57419	ASXL2ID: 55252	C14orf48 Gene ID: 256369	SLC35E1 Gene ID: 79939
BMP4 Gene ID: 652	FRMD4A Gene ID: 55691	MCF2L Gene ID: 23263	SOX4 Gene ID: 6659		C9orf57 Gene ID: 138240	SP2 Gene ID: 6668
BTAF1 Gene ID: 9044	GLDC Gene ID: 2731	MLF2 Gene ID: 8079	SULT1A2 Gene ID: 6799		CASP1 Gene ID: 834	STAT2 Gene ID: 6773
RPAP2 Gene ID: 79871	GPR107 Gene ID: 57720	MMP14 Gene ID: 4323	TAF6 Gene ID: 6878		CCNK Gene ID: 8812	TDE1 Gene ID: 10955
MIS18A Gene ID: 54069	H2AFX Gene ID: 3014	MSX2 Gene ID: 4488	TBC1D13 Gene ID: 54662		COX15 Gene ID: 1355	TIAL1 Gene ID: 7073
CENPM Gene ID: 79019	HEMK1 Gene ID: 51409	NF1 Gene ID: 4763	TBL2 Gene ID: 26608		ETS2 Gene ID: 2114	TNFRSF1BGene ID: 7133
FAM134A Gene ID: 79137	ZFP276 Gene ID: 92822	NUP62 Gene ID: 23636	TFR2 Gene ID: 7036		FREM1 Gene ID: 158326	TOE1 Gene ID: 114034
C7orf25 Gene ID: 79020	HNRNPC Gene ID: 3183	P2RY13 Gene ID: 53829	TGOLN2 Gene ID: 10618		GGA2 Gene ID: 23062	TUBD1 Gene ID: 51174
CDC20 Gene ID: 991	HNRPR Gene ID: 10236	PCSK5 Gene ID: 5125	TPM4 Gene ID: 7171		GRCC10 Gene ID: 113246	ZNF652 Gene ID: 22834
CPS1 Gene ID: 1373	HSD3B2 Gene ID: 3284	PDCD4 Gene ID: 27250	TRAF4 Gene ID: 9618		H41Gene ID: 55573	USP42 Gene ID: 84132
DDX1 Gene ID: 1653	HSPC047 Gene ID: 29060	PDXK Gene ID: 8566	TUBB Gene ID: 203068		KCNJ2 Gene ID: 3759	USP48 Gene ID: 84196

(*Continued*)

Kim *et al.*				Agostini *et al.*	Rimkus *et al.*	
EIF4A1 Gene ID: 1973	HSPC152 Gene ID: 51504	PHF7 Gene ID: 51533	TYMS Gene ID: 7298		KPNA6 Gene ID: 23633	VPS37C Gene ID: 55048
ELAVL1 Gene ID: 1994	HTR2C Gene ID: 3358	PIP3-EGene ID: 26034	UBR2 Gene ID: 23304		KPNB1 Gene ID: 3837	WTAP Gene ID: 9589
ENO1 Gene ID: 2023	IGLC2 Gene ID: 3538	PLCE1 Gene ID: 51196	ULK4 Gene ID: 54986		LOC199899 Gene ID: 199899	YY1AP1 Gene ID: 55249
ZNF84 Gene ID: 7637	ILF3 Gene ID: 3609	PYCARD Gene ID: 29108	USP14 Gene ID: 9097		MAFB Gene ID: 9935	ZNF24 Gene ID: 7572
EPS15 Gene ID: 2060	ITM2A Gene ID: 9452	RAD23B Gene ID: 5887	YIPF2 Gene ID: 78992		MFN1 Gene ID: 55669	
EZH1 Gene ID: 2145	KCNH2 Gene ID: 3757	RAP1A Gene ID: 5906			MGC14288 Gene ID: 84987	
FBS1 Gene ID: 26232	LEMD3 Gene ID: 23592	RAPGEF2 Gene ID: 9693			M-RIP Gene ID: 23164	
FGFR4 Gene ID: 2264	LMNB2 Gene ID: 84823	RPL10L Gene ID: 140801			NARF Gene ID: 26502	
FHL1 Gene ID: 2273	LOC283768 Gene ID: 283768	RPS2Gene ID: 6187			PAPD1 Gene ID: 55149	

Supplementary Table S2: Single matrix of 277 differently expressed genes obtained through *de-novo* integration of gene expression data

ProbeID				
	GC01M020174_at	GC04M149219_at	GC0XP102437_at	GC17M015073_at
	GC01M047421_at	GC04M157902_at	GC0XP115481_at	GC17M023108_at
	GC01M103055_at	GC04M169375_at	GC0YM020326_at	GC17M036285_at
	GC01M107915_at	GC04P074845_at	GC0YP002769_at	GC17M036332_at
	GC01M109988_at	GC04P075470_at	GC0YP013454_at	GC17M037029_at
	GC01M120003_at	GC04P078623_at	GC0YP020142_at	GC17M044158_at
	GC01M149603_at	GC04P089115_at	GC0YP021075_at	GC17P001612_at
	GC01M151629_at	GC04P156807_at	GC10M069226_at	GC17P007151_at
	GC01M153424_at	GC04P157044_at	GC10M069713_at	GC17P029636_at
	GC01M184907_at	GC04P166519_at	GC10M095341_at	GC17P055582_at
	GC01M201414_at	GC05M000848_at	GC10M133631_at	GC18P027281_at
	GC01M205168_at	GC05M016527_at	GC10P005126_at	GC18P059297_at
	GC01M224140_at	GC05M042835_at	GC10P029006_at	GC19M006628_at
	GC01M233846_at	GC05M095751_at	GC10P070518_at	GC19M017374_at
	GC01P000938_at	GC05M134934_at	GC10P085944_at	GC19M041087_at
	GC01P022852_at	GC05M138752_at	GC10P088718_at	GC19M045045_at
	GC01P078858_at	GC05M147184_at	GC10P091142_at	GC19M046869_at
	GC01P078888_at	GC05M151021_at	GC10P124310_at	GC19M056153_at
	GC01P086646_at	GC05P080564_at	GC11M002113_at	GC19P018358_at
	GC01P086724_at	GC05P082804_at	GC11M005203_at	GC19P050663_at
	GC01P100897_at	GC05P096238_at	GC11M046834_at	GC20M023723_at
	GC01P191044_at	GC05P137829_at	GC11M059376_at	GC20M043960_at
	GC01P204484_at	GC05P147423_at	GC11M101896_at	GC20M049646_at
	GC01P205137_at	GC05P149320_at	GC11M102146_at	GC20P009024_at
	GC01P205561_at	GC06M109414_at	GC11M102165_at	GC20P025329_at
	GC01P210805_at	GC06M116546_at	GC11M102211_at	GC20P029656_at
	GC02M079165_at	GC06M133044_at	GC11M102238_at	GC20P034603_at
	GC02M079296_at	GC06M169433_at	GC11M110728_at	GC20P043236_at
	GC02M088261_at	GC06P032434_at	GC11M113947_at	GC20P043532_at
	GC02M162707_at	GC06P046869_at	GC11P001064_at	GC20P044771_at
	GC02M183406_at	GC06P057145_at	GC11P001200_at	GC20P055569_at
	GC02M189604_at	GC07M016467_at	GC11P005667_at	GC20P060918_at
	GC02M199842_at	GC07M023316_at	GC11P057121_at	GC21M042605_at
	GC02M227935_at	GC07M027147_at	GC11P060016_at	GC21M042655_at
	GC02M237897_at	GC07M027168_at	GC11P063060_at	GC21P041720_at

(*Continued*)

ProbeID				
	GC02P017643_at	GC07M037912_at	GC11P068208_at	GC21P046342_at
	GC02P037483_at	GC07M045918_at	GC11P069604_at	GC22M022706_at
	GC02P044414_at	GC07M086970_at	GC11P116575_at	GC22P020714_at
	GC02P074031_at	GC07M099402_at	GC12M007963_at	GC0XP099705_at
	GC02P079201_at	GC07M100662_at	GC12M014926_at	GC16P088214_at
	GC02P089024_at	GC07M107193_at	GC12M045755_at	GC04M075202_at
	GC02P101974_at	GC07P022732_at	GC12M051126_at	GC04M077112_at
	GC02P108272_at	GC07P023252_at	GC12M089999_at	GC04M077132_at
	GC02P151922_at	GC07P093861_at	GC12M090041_at	GC04M077145_at
	GC02P169629_at	GC07P100302_at	GC12M115961_at	GC04M084305_at
	GC02P189547_at	GC07P133862_at	GC12P007038_at	GC04M088696_at
	GC02P217206_at	GC08M006769_at	GC12P012778_at	GC04M100457_at
	GC02P228386_at	GC08M006900_at	GC12P015366_at	GC0XM003220_at
	GC02P238060_at	GC08M027724_at	GC12P020859_at	GC0XM008456_at
	GC03M016333_at	GC08M042151_at	GC12P068028_at	GC0XM015489_at
	GC03M050312_at	GC08M095330_at	GC12P070121_at	GC0XM072957_at
	GC03M058153_at	GC08M120638_at	GC13M076352_at	GC0XM150873_at
	GC03M121595_at	GC08P024297_at	GC13P052500_at	GC0XP043400_at
	GC03M140718_at	GC08P039891_at	GC13P099539_at	GC0XP051503_at
	GC03M151165_at	GC08P070541_at	GC14M024170_at	GC16M055258_at
	GC03M159897_at	GC08P086563_at	GC14M024348_at	GC16M063538_at
	GC03M166179_at	GC08P098950_at	GC14M050441_at	GC16P000751_at
	GC03M196963_at	GC08P142501_at	GC14M093914_at	GC16P011966_at
	GC03M198162_at	GC09M036204_at	GC14M103055_at	GC16P054070_at
	GC03P062279_at	GC09M074705_at	GC14M104476_at	GC16P055216_at
	GC03P123526_at	GC09M094258_at	GC14M105876_at	GC16P065525_at
	GC03P150065_at	GC09M116822_at	GC14P030413_at	GC04M069085_at
	GC03P161881_at	GC09P006206_at	GC14P074815_at	GC04M071761_at
	GC03P185762_at	GC09P033230_at	GC15M043172_at	GC04M075086_at
	GC04M015578_at	GC09P035663_at	GC15P030797_at	GC04M075092_at
	GC04M056156_at	GC09P074956_at	GC15P043331_at	GC04M075100_at
	GC04M057210_at	GC09P100746_at	GC15P057691_at	GC09P129951_at
	GC09P134927_at	GC15P072253_at	GC16M045672_at	
	GC09P134947_at	GC15P078858_at	GC0XM002846_at	
	GC16M014673_at	GC16M028457_at	GC09P136673_at	

Supplementary Table S3: Significantly enriched pathways and *p*-values for: (a) figure 2 and (b) figure 4

a) Pathway	*p*-value
Immune System	3,64E-07
Signal Transduction	0,024148
Hemostasis	0,181925
Metabolism	0,21381
Apoptosis	0,220759
Developmental Biology	0,370575

b) Pathway	*p*-value
Immune System	3,14E-16
RNF125 mediated ubiquitination of RIG-I, MDA5 and IPS-1	6,41E-07
Hemostasis	9,07E-06
Signal Transduction	0,002719
Metabolism	0,002898
APC-Cdc20 mediated degradation of Nek2A	0,008458
Cell-Cell communication	0,013402
Gene Expression	0,023573
Apoptosis	0,053735
Cdc20:Phospho-APC/C mediated degradation of Cyclin A	0,059329
Disease	0,070436
Developmental Biology	0,143688
Cell Cycle	0,148407
Neuronal System	0,166389
Muscle contraction	0,235869
Metabolism of RNA	0,338982
DNA Repair	0,345511
DNA Replication	0,353839

Supplementary Table S4: Raw data obtained from "The Comparative Toxicogenomics Database" (CTD) for figure 3A. Data for figure 3B is extracted from the network used to produce figure 3A.
